# Food allergens in oral care products

**DOI:** 10.1038/s41598-023-33125-y

**Published:** 2023-04-24

**Authors:** Luísa Coimbra, Isabel Margarida Costa, José Grillo Evangelista, Alexandra Figueiredo

**Affiliations:** 1 Instituto Universitário Egas Moniz (IUEM), Campus Universitário-Quinta da Granja, 2829-511 Monte de Caparica, Portugal; 2 PharmSci Lab/Centro de Investigação Interdisciplinar Egas Moniz (CiiEM), Campus Universitário-Quinta da Granja, 2829-511 Monte de Caparica, Portugal; 3 Morphology Lab/Centro de Investigação Interdisciplinar Egas Moniz (CiiEM), Campus Universitário-Quinta da Granja, 2829-511 Monte de Caparica, Portugal

**Keywords:** Health care, Dentistry

## Abstract

Food allergies are a growing concern, especially in Western societies and can dramatically impact the quality of life of affected individuals. In recent years, various food allergens have been introduced into the oral care industry to improve product properties and provide the best possible treatment. Since small doses of food allergens may be sufficient to trigger allergic reactions, the non-discrimination of the sources of certain excipients in the product composition can compromise the patient's health. Therefore, knowledge and awareness of allergies and product composition among health professionals are critical on behalf of patients’ and consumers’ health. This study aimed to ascertain the presence of dairy products (e.g., cow's milk proteins and lactose), cereals (e.g., gluten, soy, and oats), fruits, nuts, spices, shellfish, and additives as excipients in oral care products for outpatients and products for professional use in the Dental Office. Among the 387 surveyed products, the highest prevalence of food allergens was found in toothpaste, fluoride varnishes, and alginates, mostly in spices and fresh fruits. As food allergies may occur because of erroneous information or a lack of labeling on the allergen list, manufacturers should be more rigorous in declaring allergens on product labeling regarding the safety of consumers.

## Introduction

In recent decades, the impact of food allergies on humanity has been remarkable, and it continues to be a common and relevant topic for investigations globally^1^. Food allergies affect approximately 5% of adults and 8% of children^2^. It is estimated that 220 million people worldwide have allergic reactions to certain foods^3^. In the United States, 1–10% of the population suffers from allergic diseases^4,5^, and Nwaru et al.^6^ found in a systematic review that the same upward trend has been reported in Europe, with prevalence ranging from 1 to 6%, with cow's milk protein (CMP) and egg allergies being the most common among children.

According to the definition provided by the National Institute of Allergy and Infectious Diseases (NIAID) in 2010^7^, food allergy constitutes an "adverse health effect arising from a specific immune response, which occurs reproducibly upon exposure to a particular food". Allergies can be immunoglobulin E (IgE)-mediated, non-IgE-mediated, or mixed^8^. An IgE-mediated immune reaction is characterized by the onset of symptoms within minutes of interaction with the allergen, and is the most life-threatening^9,10^. Non-IgE-mediated immune reactions occur when there is an interaction between an allergen and the gastrointestinal tract with a delayed onset of symptoms^11^. Allergic reactions can affect the entire body, with skin, respiratory, cardiovascular, gastrointestinal, or even neurological manifestations. Symptoms can range from mild to moderate (hives, cough, swelling of the face, tongue, or lips, abdominal pain, skin rash, tingling sensation, hoarse throat, difficulty swallowing, vomiting, and diarrhea) or severe (difficulty breathing, dizziness, loss of consciousness, low blood pressure, cardiac arrest, and anaphylactic reactions)^12,13^.

The current approach to allergy management is largely based on prevention of allergic reactions. Treatment principles require extensive patient education, and in the case of drugs or clinically applied products, a high level of knowledge and awareness from health professionals^2^.

The oral cavity is continuously exposed to a wide spectrum of antigens, including dental materials, oral hygiene products, medicines, and foods. Therefore, although complaints of adverse reactions are uncommon, patients may be in contact with many allergens^14^. Several food allergens have been introduced into the oral care industry to improve their properties and provide the best possible treatment. Even small doses of food allergens may be sufficient to trigger allergic reactions in the most sensitive patients, which makes knowledge of the composition of these products extremely important to ensure patient safety^15^.

Dentists’ knowledge of allergy triggers and attention to the composition of oral care products can help prevent possible allergies.

## Aim

This study was developed to ascertain the presence of food allergens in oral care products (OCP) used in dentistry through a label survey.

### Methodology

A total of 387 OCP labels were assessed. Products used in or with possible contact with the oral cavity, for pediatric or adult use, and those that did not require a medical prescription were eligible for inclusion. To cover as many products as possible, this study included outpatient OCP and products for professional use in the Dental Office.

Drugs and products that did not show labeling information regarding their detailed composition were excluded from the study.

The oral hygiene outpatient products chosen were:

- 8 packs of chewing gum;
- 4 denture cleaning tablets;
- 4 denture fixative creams;
- 66 types of mouthwash;
- 17 oral gels;
- 9 oral sprays;
- 4 orthodontic waxes;
- 187 toothpastes.

The Dental Office products chosen were as follows:

- 39 alginates;
- 23 fluoride varnishes;
- 6 packs of gloves;
- 6 plaque revealing cream;
- 12 prophylactic toothpastes;
- 2 topical creams.

The products were selected according to predefined inclusion and exclusion criteria. The research was conducted by reading the ingredients on the product labels to identify substances with a potential allergenic nature. The WHO and EFSA official documents concerning the evaluation of allergenic foods and food ingredients were used as references for the selection of excipients covered in this study^16,17^. The compounds searched were cereals, dairy products (cow milk proteins, and lactose), food additives, fruits, nuts, shellfish, and spices.

## Results and discussion

This survey revealed a 46.3% prevalence of possible food allergens in the studied OCP (179 out of 387 products). Most of the samples (81%) contained only one food allergen. However, its stand out a toothpaste with a total of 5 different allergens.

The presence of food allergens as excipients was common in both outpatient and Dental Office products. In outpatient products these allergens were predominant in toothpaste, chewing gum, and orthodontic waxes; in Dental Office products, the highest reference to food allergens was found in topical creams, fluoride varnishes, alginates, prophylactic toothpaste and gloves (Table 1). It should be noted that the absence of references to food allergens in the list of excipients does not eliminate the possibility of their presence.

**Table 1:** Prevalence of food allergens in the labeling.

	N (total)	Food allergen as excipient (%)
Outpatient products		
Chewing gum	8	6 (75.0%)
Denture cleaning tablets	4	0
Denture fixative creams	4	0
Mouthwashes	66	20 (30.3%)
Oral gels	17	5 (29.4%)
Oral sprays	9	1 (11.1%)
Orthodontic waxes	4	3 (75.0%)
Toothpaste	187	86 (46.0%)
Dental office products		
Alginates	39	22 (56.4%)
Fluoride varnishes	23	21 (91.3%)
Gloves	6	4 (66.7%)
Plaque revealing cream	6	1 (16.7%)
Prophylactic toothpaste	12	8 (66.7%)
Topical creams	2	2 (100%)

The most frequently mentioned types of food allergens were fruits and spices. The labelling of denture fixative creams and denture cleaning tablets does not refer to any type of food allergen (Table 2).

**Table 2:** Different types of food allergens mentioned in the oral care product labeling.

	Cereals	Dairy products	Food additives	Fruits	Nuts	Shellfish	Spices
Outpatient products							
Chewing gum	4	–	5	–	1	–	1
Denture fixative creams	–	–	–	–	–	–	–
Denture cleaning tablets	–	–	–	–	–	–	–
Mouthwashes	–	–	–	13	–	1	14
Oral gels	1	1	–	–	–	1	2
Oral sprays	–		–	–	–	–	1
Orthodontic waxes	–	1	–	2	–	–	–
Toothpaste	6	3	1	42	1	–	71
Dental office products							
Alginates	–	–	–	16	–	–	6
Fluoride varnishes	–	4	–	19	–	–	1
Gloves	4	–	–	–	–	–	–
Plaque revealing cream	–	1	–		–	–	–
Prophylactic toothpaste	–	1	–	7	–	–	–
Topical creams	–	2	–	–	–	–	2

### Cereals

Most cereals belong to the *Gramineae* family with a high degree of IgE cross-reactivity between allergens from cereal seeds and grass pollen. Wheat is the predominant cereal that causes food allergies in humans^17^ and is responsible for several disorders, such as celiac disease, wheat allergy, and non-celiac gluten sensitivity. These diseases have a prevalence of 1–6% in Europe and North America^18^. Gluten is an increasingly used component in dental and oral hygiene materials due to the demand for biodegradable yet resilient materials^19^.

A clinical case was reported in a 9-year-old patient with celiac disease, who constantly manifested symptoms of active disease despite following a restricted gluten-free diet. Adverse reactions were associated with the daily use of an orthodontic retainer composed of plasticized methacrylate polymer and gluten. Gluten is often incorporated in plastics as an additive^15^.

Knowledge of food allergies implies being aware of the possibility of cross-reactivity between different allergens. Gluten may be present even in trace amounts in several other compounds. Maltodextrin is derived from corn or wheat starch. However, it is highly processed, with gluten eventually being removed almost entirely, which allows it to be considered scientifically gluten-free and therefore safe for patients with celiac disease^20^. In this survey, maltodextrin was mentioned on the labels of one oral gel and six toothpastes (Table 2). However, the exact source of this compound was not indicated on the products labels.

According to Hall et al.^19^, there is a need for more restrictive legislation for labeling the gluten content of non-food products, as there is in food and pharmaceutical products. The lack of legislation, coupled with the fact that research addressing gluten in oral products is scarce, leads to potential chronic low-level gluten intake in patients with celiac disease, who are unaware of the presence of this compound in OCP^19^.

Oats are often contaminated with gluten as they are grown and processed in the same places as wheat, rye, and barley. Thus, a celiac who can eat oats should always look for a "gluten-free" designation to ensure that it is free of contamination^18^. Only 5% of patients with celiac disease are allergic to oats. These individuals react to a specific protein in oats to which they are intolerant, called avenin. In the other 95% of patients with celiac disease, oats can be carefully introduced with close monitoring of symptoms and frequent laboratory testing. In the case of celiac patients who are intolerant to avenin, the consumption of oats is not advised because the problem lies in the presence of this protein and not the possible contamination^21^.

Oats were listed on the labels of four glove packages. The lack of data on the origin of oats prevents a final decision on whether these gloves should be used in patients with celiac disease.

In the present study, soy appeared as an excipient in four chewing gum samples. Soy contains approximately 28 allergenic proteins. Although soy allergy symptoms are generally minor, severe symptoms and anaphylaxis following oral exposure to soy have also been reported^22^.

### Dairy products

This study also revealed that dairy products may be present in various sample types. Dairy excipients found include lactose and cow milk proteins, such as casein and lactoferrin. Since caramel and chocolate flavorings may contain milk derivatives in their composition, they were also included. Caramel flavoring was the most prevalent dairy allergen, followed by casein (Table 3).

**Table 3:** Dairy allergens in the studied OCP labeling.

	Alginates	Chewing gum	Fluoride varnishes	Gloves	Mouthwashes	Oral gels	Oral spray	Orthodontic waxes	Plaque revealing cream	Prophylactic toothpastes	Toothpastes	Topical cream
Cow milk proteins												
* Casein*	–	–	1	–	–	–	–	–	–	–	–	2
* Lactoferrin*	–	–	–	–	–	–	–	–	–	–	1	–
Milk protein*	–	–	–	–	–	1	–	–	–	–	–	–
Lactose	–	–	–	–	–	–	–	–	1	–	1	–
Chocolate	–	–	–	–	–	–	–	1	–	–	–	–
Caramel	–	–	3	–	–	–	–	–	–	1	1	–

*No specification on the label of which milk protein was used.

Cow's milk protein allergy (CMPA) is the most prevalent food allergy in infants and children, and results from an immune reaction that develops in the first year of life. Patients with CMPA should avoid the consumption of milk or dairy products in the diet of the child and mother.

The lactose used as an excipient undergoes purification to remove CMP, making it safe for patients with CMP^23^. However, cross-contamination by proteins can occur during this process, resulting in allergic reactions. Some examples have been reported in the literature, namely, in the pediatric population after treatment with drugs containing lactose with traces of CMP^24^.

According to Fiocchi et al.^25^, many manufacturers prefer to use lactose extracted from milk rather than synthetic lactose because of its high cost. Thus, lactose produced after the refinement and manufacturing processes of dairy products may contain a fraction of CMP, even if minimal^25^. To avoid unwanted allergic reactions, clinicians recommend that individuals with CMPA avoid foods containing lactose due to the risk of cross-contamination with milk proteins^26^.

### Food additives

Food additives can be used as colorants, preservatives, emulsifiers, thickening agents, and flavor enhancers. Guar gum (E412) was found in one toothpaste label, and arabic gum (E414) was an excipient in five of the surveyed chewing gums. Its use has been associated with the triggering of several clinical symptoms that, although generally rare, may include serious reactions, including anaphylactic reactions^27^.

### Fruits

Oral allergy syndrome, also known as pollen-food allergy syndrome or pollen-related food allergy, is a relatively common allergic reaction caused by cross-reacting homologous proteins found in plant foods and pollen^28,29^. The most common symptoms are itchy lips, throat, gingiva, mouth, and/or tongue. Dentists may suspect oral syndrome in patients who report oral inflammation immediately after contact with specific fruits, nuts, vegetables, and spices^28,29^. A study performed in England with 274 pollen-allergic adults revealed that 34% were sensitive to apples, 22% to peaches, and 16% to melons. In Sweden, most pollen-allergic adults are allergic to hazelnuts, apples, and peanuts. In Spain, peaches are the most common fruits which causes allergy^28^.

In the present survey, fruit essences were mentioned in alginates, fluoride varnishes, mouthwashes, orthodontic waxes, prophylactic toothpastes, and mostly in toothpastes, as shown in Table 2.

The list of fruits mentioned in the oral care product labels is presented in Table 4.

**Table 4:** Fruits in the studied OCP labeling.

	Alginates	Chewing gum	Fluoride varnishes	Gloves	Mouthwashes	Oral gels	Oral spray	Orthodontic waxes	Plaque revealing cream	Prophylactic toothpastes	Toothpastes	Topical cream
Apple	1	–	–	–	2	–	–	–	–	1	3	–
Apricot	1	–	1	–	–	–	–	–	–	–	–	–
Banana	–	–	–	–	–	–	–	–	–	–	1	–
Cherry	–	–	4	–	–	–	–	–	–	2	1	–
Citrus	1	–	–	–	–	–	–	–	–	–	–	–
Citrus grandis (grape)	–	–	1	–	1	–	–	–	–	–	4	–
Citrus medica limonum (lemon)	–	–	–	–	–	–	–	–	–	–	1	–
Cocos nucifera (coconut)	–	–	–	–	1	–	–	–	–	–	6	–
Fruit	1	–	–	–	–	–	–	–	–	–	–	–
Grapefruit		–	–	–	1	–	–	–	–	–	1	–
Kiwi	–	–	–	–	–	–	–	–	–	–	1	–
Lycium barbarum (goji)	–	–	–	–	1	–	–	–	–	–	–	–
Mango	1	–	–	–	–	–	–	–	–	–	–	–
Melon	–	–	6	–	–	–	–	–	–	–	–	–
Orange	2	–	1	–	–	–	–	–	–	2	1	–
Papaya	–	–	–	–	1	–	–	–	–	–	4	–
Peach	1	–	1	–	–	–	–	–	–	–	1	–
Pear	–	–	–	–	1	–	–	–	–	–	–	–
Pineapple	–	–	2	–	–	–	–	2	–	–	3	–
Prunus spinosa (sloe)	–	–	–	–	–	–	–	–	–	–	1	–
Punica granatum (pomegranate)	–	–	–	–	1	–	–	–	–	–	3	–
Raspberry	2	–	2	–	–	–	–	–	–	–	1	–
Red fruits	–	–	–	–	1	–	–	–	–	1	–	–
Strawberry	2	–	1	–	1	–	–	–	–	1	6	–
Tropical Fruits	2	–	–	–	–	–	–	–	–	–	–	–
Tutti frutti	2	–	–	–	–	–	–	–	–	–	–	–
Vaccinium macrocarpon (cranberry)		–	–	–	2	–	–	–	–	–	1	–
Vaccinium myrtillus (blueberry)	–	–	–	–	–	–	–	–	–	–	1	–
Watermelon	–	–	–	–	–	–	–	–	–	–	1	–

Coconut (N = 7) and strawberry (N = 11) were the most prevailing fruits in the oral care products surveyed. Although coconut reactions are sporadic, the presence of coconut should not be neglected since there is evidence of allergy to this fruit, which tends to provoke systemic responses, such as anaphylaxis^30^. Strawberry is a fruit that does not cause many allergic reactions. However, when it occurs, it is mainly as cutaneous reactions, since strawberries cause histamine release^31^.

Peach and melon, which are the fruits most responsible for allergic reactions in Portugal^31^, were found in several of the studied products. Melon was the most prevalent allergen in fluoride varnishes, while peaches were found in alginates, fluoride varnishes, and toothpaste (Table 4).

Grape, pomegranate, cherry, pineapple, orange, watermelon, and citrus fruits can also cause allergic reactions, albeit less frequently^28^, and were found in several labels of the studied samples.

### Nuts

Allergy to nuts (tree nuts and peanuts) is one of the most common food allergic reactions^10^. Although, nuts were only mentioned in two products (one chewing gum and one toothpaste), as indicated in Table 2.

### Shellfish

Shellfish allergies are relatively common, affecting around 0.5–2.5% of the world's population^32^. Chitosan is the main component of the exoskeletons of crustaceans, mollusks, and cephalopods. It may contain residues of tropomyosin, a protein that is considered highly allergenic and the European Food Safety Authority—EFSA (2014) suggests that it is the main contributor to shellfish allergy^17^. Although chitosan was only found as an excipient in two of the studied samples (one mouthwash and one oral gel), a susceptible individual when ingesting shellfish can suffer from flushing of the face, nausea, vomiting, sneezing, difficulty breathing, hypotension, and even anaphylaxis, which can be fatal^33^.

### Spices

Spices are used as ingredients in numerous products, such as peppermint or cinnamon oil used to flavor toothpaste, dental products, and alcoholic drinks^34^. In our study, spices were found in most oral care products, with the exception of orthodontic waxes, plaque-revealing creams, and prophylactic toothpastes (Table 2). The different spices found in the studied products are shown in Table 5.

**Table 5:** Spices in the studied OCP labeling.

	Alginates	*Chewing gum*	*Fluoride varnishes*	*Gloves*	*Mouthwashes*	*Oral gels*	*Oral spray*	*Orthodontic waxes*	*Plaque revealing cream*	*Prophylactic toothpastes*	*Toothpastes*	*Topical cream*
*Basil*	–	–	–	–	–	–	–	–	–	–	1	–
*Cinnamomum Zeylanicum Bark (cinnamon)*	–	–	–	–	–	–	–	–	–	–	5	–
*Curcuma xanthorrhiza (turmeric)*	–	–	–	–	1	–	–	–	–	–	–	–
*Eugenia caryophyllus (clove)*	–	–	–	–	–	–	–	–	–	–	5	–
*Foeniculum vulgare (fennel)*	–	–	–	–	2	–	–	–	–	–	5	–
*Mentha piperita (peppermint)*	2	–	1	–	6	1	1	–	–	–	42	–
*Panax ginseng (ginseng)*	–	–	–	–	1	–	–	–	–	–	–	–
*Parsley*	–	1	–	–	–	–	–	–	–	–	4	–
*Piper nigrum (black pepper)*	–	–	–	–	–	–	–	–	–	–	1	–
*Rosmarinus officinalis (rosemary)*	–	–	–	–	1	1	–	–	–	–	3	–
*Thymus vulgaris (thyme)*		–	–	–	1	–	–	–	–	–	–1	–
*Vanilla*	–4	–	–	–	–	–	–	–	–	–	1	2
*Zinziber officinale (ginger)*		–	–	–	2	–	–	–	–	–	3	–

The high prevalence of *Mentha piperita* (peppermint) in toothpastes and mouthwashes stands out. Scientific literature reports multiple cases of peppermint-related skin and respiratory reactions^35,36^.

Denaxa and Arwright^37^ recently reported an allergic reaction to fennel toothpaste in an 11-year-old child who tested positive for serum IgE to this substance. Fennel was reported on the labels of five toothpastes and one mouthwash^37^.

In another case, an anaphylactic reaction induced by a herbal medicine confirmed the allergenic content of ginger^38^. Ginger (*Zinziber officinale*) was an excipient in three toothpastes, and two mouthwashes (Table 5).

Additionally, other plants found in the surveyed products had a history of allergic reactions, including rosemary^39,40^, clove^41^, parsley^42^, and saffron^43^.

Graphs showing the allergens present in the different product groups are available in Supplementary Figures 1.

## Conclusions

The dentist is responsible for ensuring that the best oral healthcare is provided. Knowledge of the composition of OCP and their adaptation to patients' pathophysiological conditions is fundamental for safe and effective treatment. To achieve this goal, product labels should detail their composition, and clinicians should be alert to food allergies, be aware of the risks to the patient's health, and include questions about food allergies in their clinical history to make a conscious and careful selection of products to be used by the patient.

This investigation revealed a high prevalence of excipients with the potential to trigger allergic reactions. However, the labels do not mention the amounts of these compounds and also do not always mention food sources, which is essential information that may be the deciding factor in whether to use the product. Since even small amounts of food allergens can cause allergic reactions, non-discrimination of the sources of certain excipients can jeopardize a patient’s health. Furthermore, although the labeling of the oral health products under study is clear and explicit regarding excipients, it does not always warn against the risk of food allergens.

Manufacturers should be more rigorous in declaring allergen sources and quantities on product labeling, and the urgency of highlighting the presence of allergens on labels must be emphasized to avoid adverse and potentially dangerous reactions and safeguard consumers' health.

## Supplementary Information

Supplementary Figures.

Supplementary Information.

## Data Availability

All data generated or analysed during this study are included in this published article and its supplementary information files.
