# Supplementary material for: Food allergens in oral care products
Source: Sci Rep. 2023 Apr 24;13:6684. doi: 10.1038/s41598-023-33125-y (PMC10126110; doi:10.1038/s41598-023-33125-y)
Supplement: Supplementary file 1 — Supplementary Figures. [file 41598_2023_33125_MOESM1_ESM.pdf]

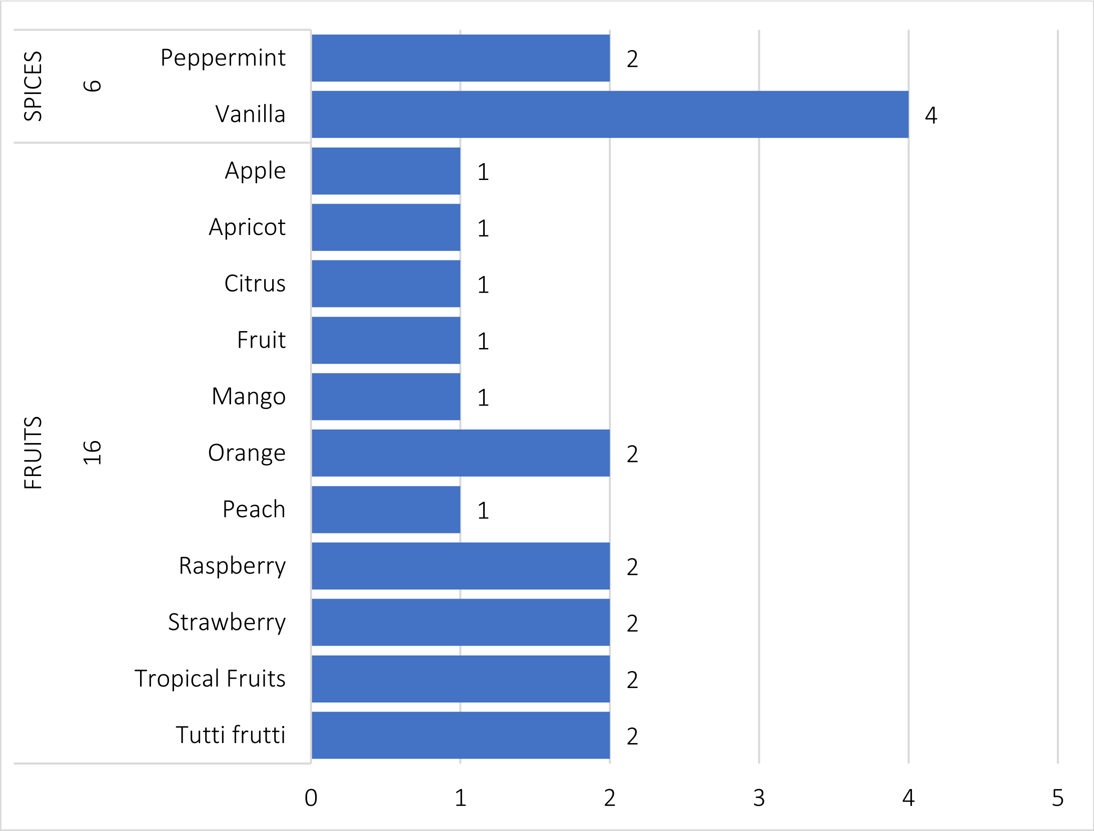

SUPPLEMENTARY FIGURE S1 - Alginates: food allergens present in the list of excipients (N)

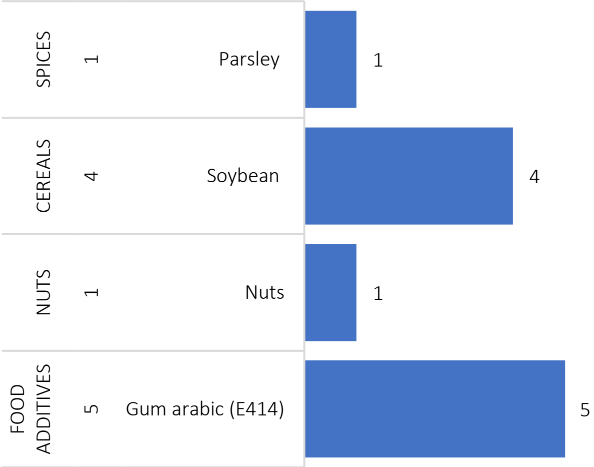

SUPPLEMENTARY FIGURE S2- Chewing Gum: food allergens present in the list of excipients (N)

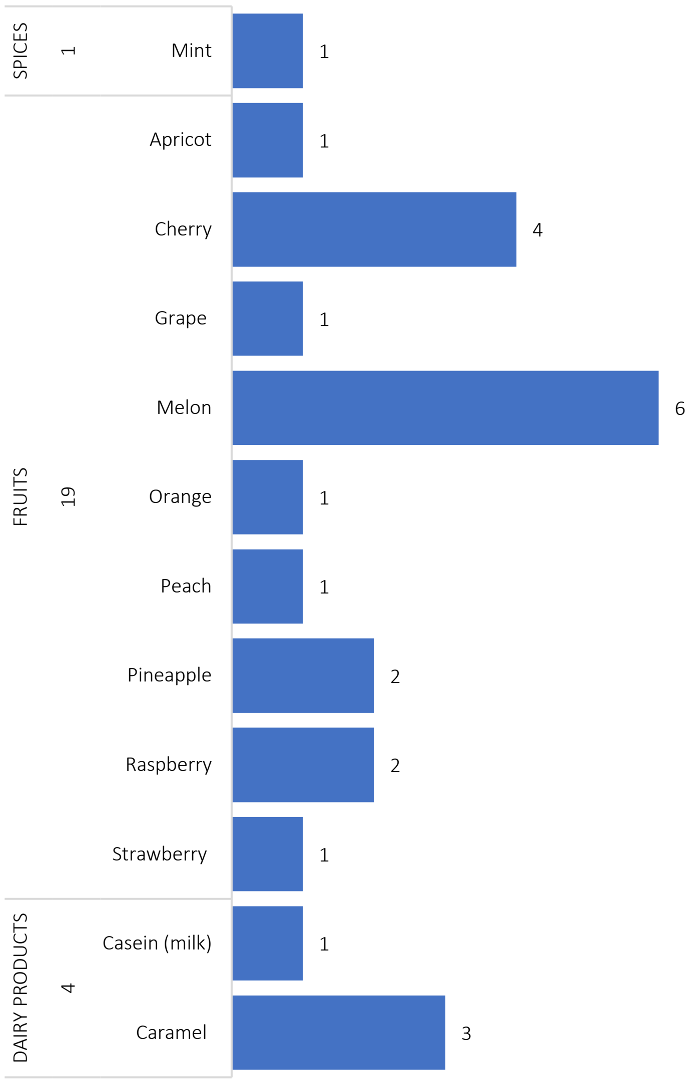

SUPPLEMENTARY FIGURE S3 - Fluoride varnishes: food allergens present in the list of excipients (N)

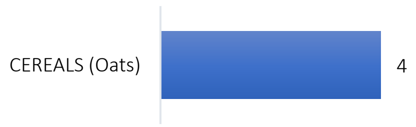

SUPPLEMENTARY FIGURE S4 - Gloves: food allergens present in the list of excipients (N)

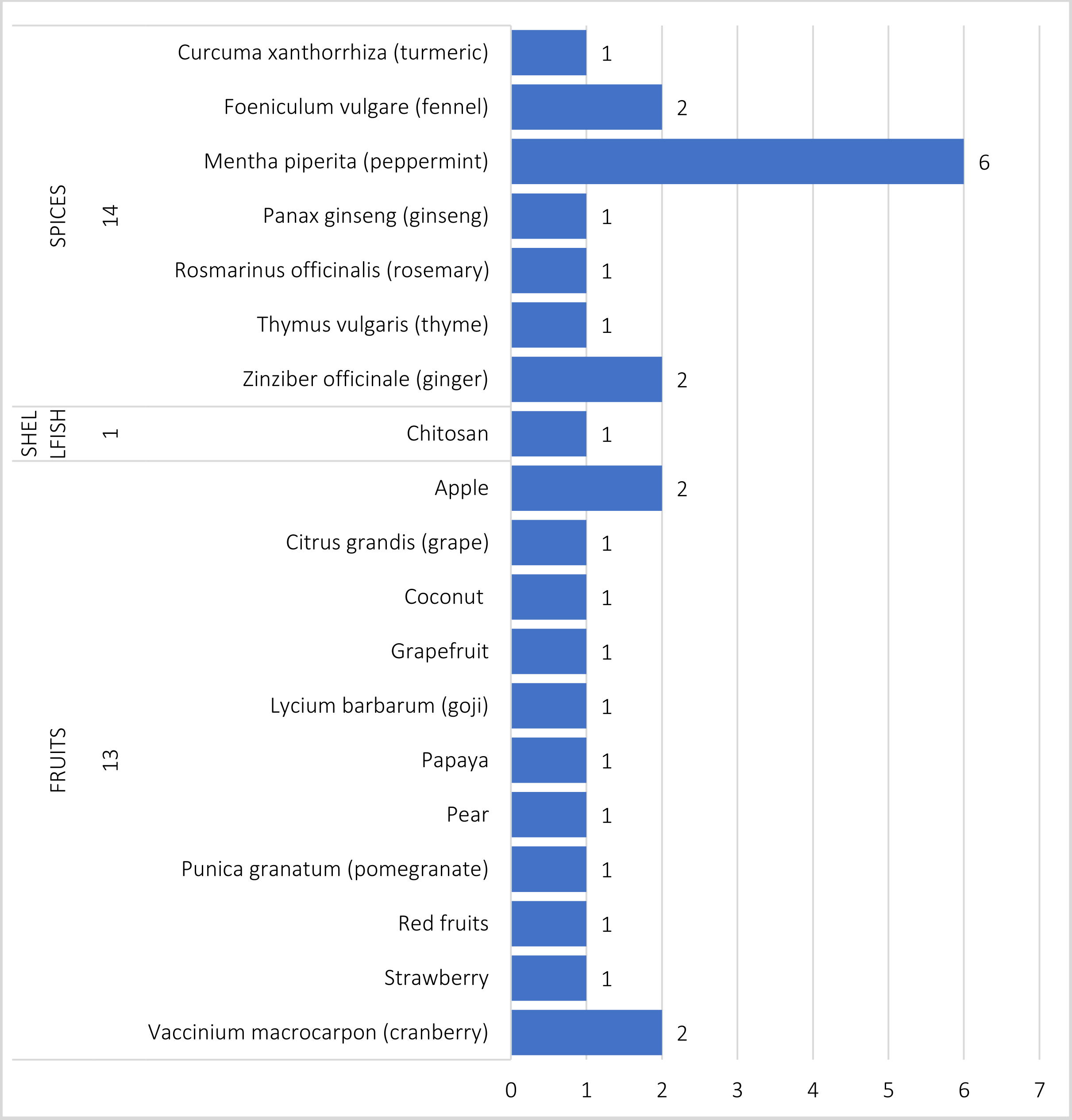

SUPPLEMENTARY FIGURE S5 - Mouthwashes: food allergens present in the list of excipients (N)

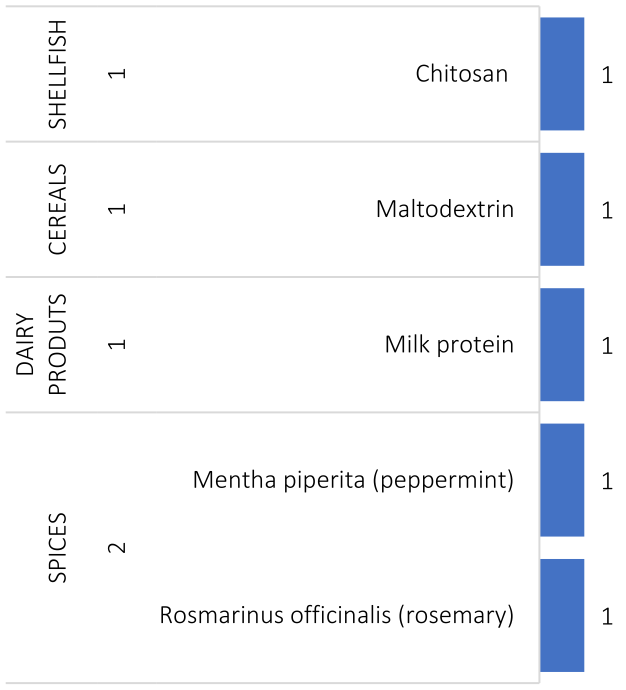

SUPPLEMENTARY FIGURE S6 - Oral Gels: food allergens present in the list of excipients (N)

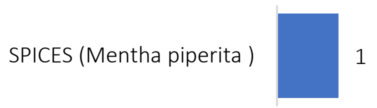

SUPPLEMENTARY FIGURE S7 - Oral spray: food allergens present in the list of excipients (N)

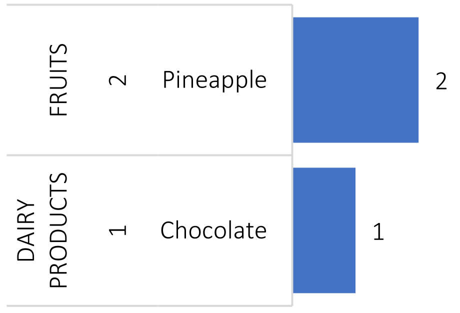

SUPPLEMENTARY FIGURE S8 - Orthodontic waxes: food allergens present in the list of excipients (N)

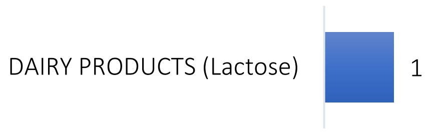

SUPPLEMENTARY FIGURE S9 - Plaque revealing cream: food allergens present in the list of excipients (N)

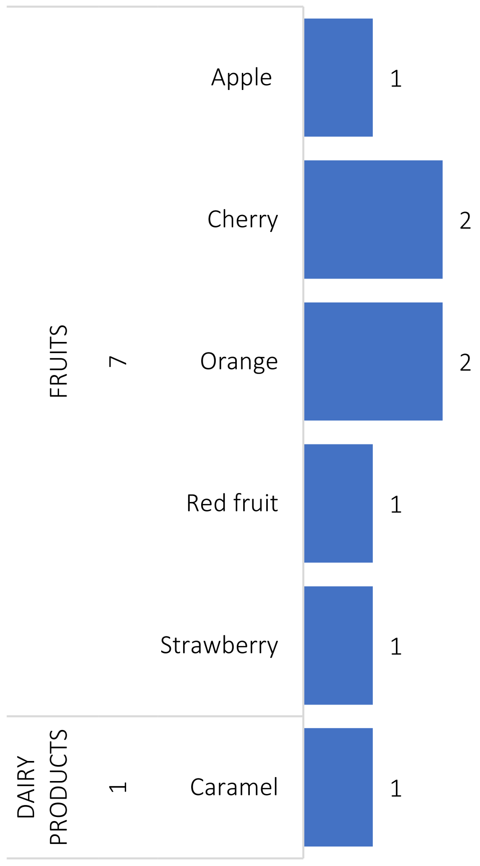

SUPPLEMENTARY FIGURE S10- Prophylactic toothpastes: food allergens present in the list of excipients (N)

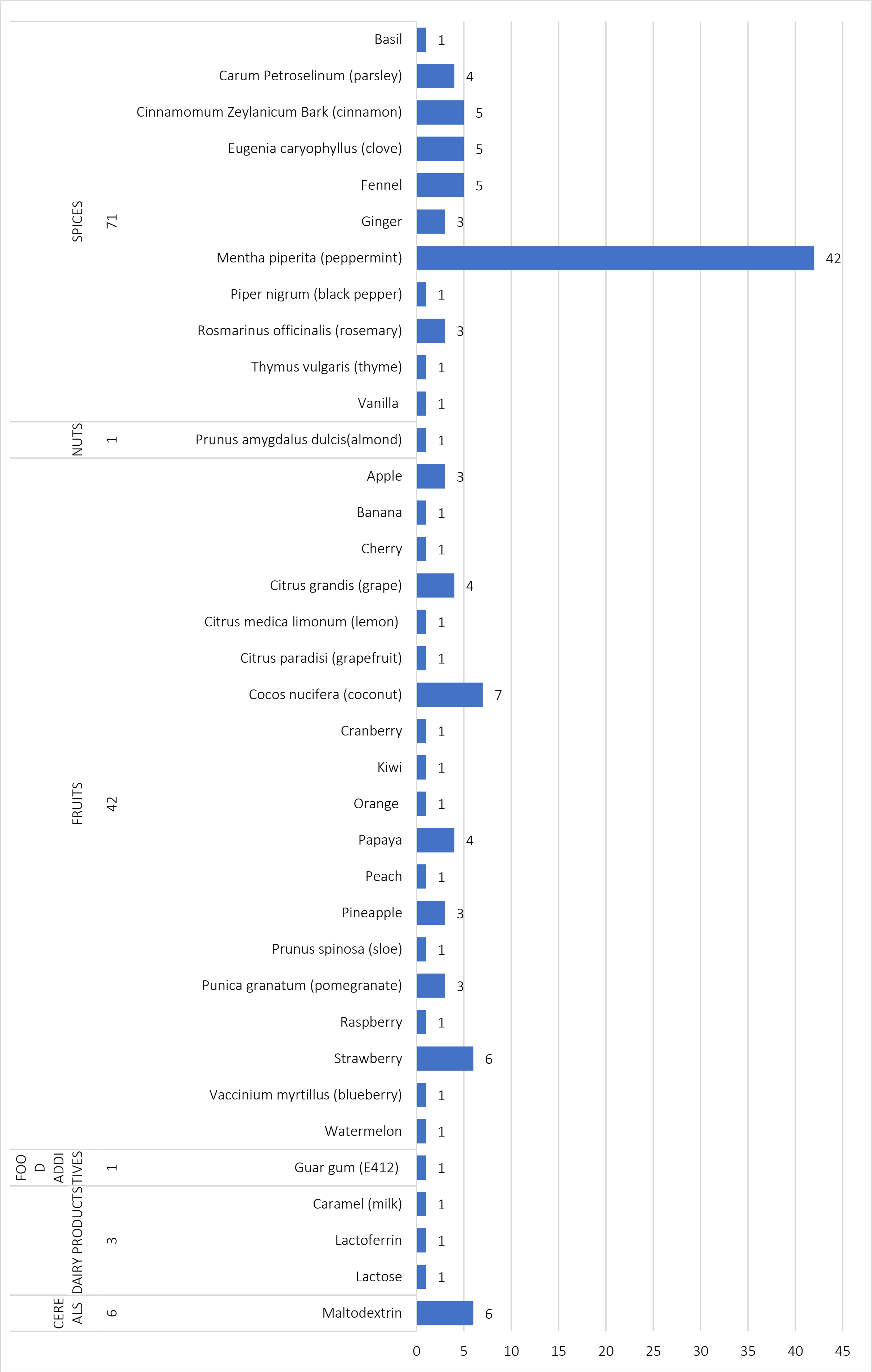

SUPPLEMENTARY FIGURE S11 - Toothpastes: food allergens present in the list of excipients (N)

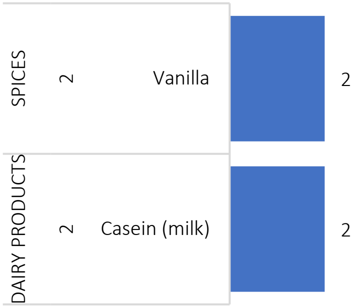

SUPPLEMENTARY FIGURE S12 - Topical creams: food allergens present in the list of excipients (N)
