# Supplementary material for: Food allergens in oral care products
Source: Sci Rep. 2023 Apr 24;13:6684. doi: 10.1038/s41598-023-33125-y (PMC10126110; doi:10.1038/s41598-023-33125-y)
Supplement: Supplementary file 2 — Supplementary Information. [file 41598_2023_33125_MOESM2_ESM.pdf]

Oral care products	BRAND	FORMULA	PRESENCE OF FOOD ALLERGEN	#
**Toothpastes**	3M - ESP	Clinpro Tooth Crème	Vanilla flavor	1
	A. Vogel	Echinacea	Essence of Mentha piperita (Peppermint)<br>Vaccinium myrtillus (bilberry) extract<br>Rosmarinus Officinalis Essence (rosemary)	2
	Aloe Dent	Triple Action Aloe Vera	Essence of Mentha piperita (Peppermint)	3
	Aloe Dent	Triple Action Coconut Oil	Essence of Cocos nucifera (coconut)<br>Essence of Mentha piperita (Peppermint)	4
	Aloe Dent	Triple Action Spearmint	Essence of Mentha piperita (mint) pepper)	5
	Aloe Dent	Triple Action Whitening	Essence of Mentha piperita (Peppermint)	6
	Aloe Dent	Triple Action Whitening Without Fluoride	Essence of Mentha piperita (Peppermint)	7
	Aquafresh	Active White		8
	Aquafresh	Advance 9 - 12 years		9
	Aquafresh	Intense white		10
	Aquafesh	Milk teeth 1 - 5 years		11
	Aquafresh	Junior 6 - 9 years old		12
	Aquafresh	Triple protection - fresh mint		13
	Aquafresh	Triple protection - caries protection		14
	Aquafresh	Triple action - eucalyptus, citron and menthe		15
	Aquafresh	Splash 3 - 8 years old	Strawberry flavor	16
	Australian Tea Tree	Whitening toothpaste Fluoride-free melaleuca	Essence of Mentha piperita (mint) pepper)	17
	Bam&Boo	Natural		18
	Bam&Boo	Toothpastes		19
	Ben&Anna	Toothpaste Natural Black		20
	Ben&Anna	Toothpaste Natural Cinnamon	Essence of Cinnamomum Zeylanicum (cinnamon)	21
	Ben&Anna	Natural Sensitive Toothpaste		22
	Biorepair	Parodontgel	Lactoferrin	23
	BlanX	Whitening toothpaste coconut	Coconut oil	24
	Buccotherm	3+ years		25
	Buccotherm	2 - 6 years	Strawberry flavor	26
	Buccotherm	7 - 12 years (peach iced tea flavor)	Caramel<br>Peach flavor	27
	Buccotherm	7 - 12 years (mint flavor)		28

	Buccotherm	Sensitive Gums Toothpaste Gel with Fluoride		29
	Buccotherm	Sensitive Gums Toothpaste Fluoride-Free Gel		30
	Buccotherm	Tooth Decay Prevention Toothpaste		31
	Buccotherm	Whitening & Care Toothpaste		32
	Buccotherm	Whitening Toothpaste with Activated Charcoal Organic Certified		33
	Cattier	Kids 2 - 6 years old raspberry	Raspberry flavor	34
	Cattier	Kids +7 years mint		35
	Cattier	Dentargile Clay + Rosemary	Rosmarinus Officinalis Essence (rosemary)	36
	Cattier	Dentargile Clay + Mint	Essence of Mentha piperita (Peppermint)	37
	Cattier	Eridène Toothpaste Fragile Gums	Maltodextrin<br>Papain<br>Bromelain (pineapple)	38
	Cattier	Eridène Toothpaste Fresh Breath 0% Peroxide	Essence of Mentha piperita (Peppermint)<br>Maltodextrin<br>Papain<br>Bromelain (pineapple)	39
	Cattier	Eridène Whitening Toothpaste Fluoride Free	Maltodextrin<br>Papain<br>Bromelain (pineapple)	40
	Colgate	Anti-Tartar + Whitening		41
	Colgate	Cavity Protection	Essence of Mentha piperita (Peppermint)	42
	Colgate	Dentagard	Essence of Mentha piperita (Peppermint)	43
	Colgate	Fresh Gel	Essence of Mentha piperita (Peppermint)	44
	Colgate	Revitalizing Gums Detox - minerals and coconut oil	Hydrogenated coconut oil	45
	Colgate	Herbal Original		46
	Colgate	Max White Clay and Minerals		47
	Colgate	Max White Coal		48
	Colgate	Max White Expert Complete		49
	Colgate	Max White One	Essence of Mentha piperita (Peppermint)	50
	Colgate	Max White Optic		51
	Colgate	Maximum Protection Caries with Acid-Sugar Neutralizer		52
	Colgate	Natural Extracts Charcoal		53
	Colgate	Periogard Plus		54
	Colgate	Protection Caries	Essence of Mentha piperita (Peppermint)	55
	Colgate	Sensitive Pro-Immediate Relief - daily protection	Essence of Mentha piperita (Peppermint)	56
	Colgate	Sensitive Pro-Release + Whitener	Essence of Mentha piperita (Peppermint)	57

	Colgate	Sensitive Pro-Release Repairs & Prevents		58
	Colgate	Sensitive Pro-Extra Strong Relief	Essence of Mentha piperita (Peppermint)	59
	Colgate	Total Original	Essence of Mentha piperita (Peppermint)	60
	Colgate	Total Advanced Visible Action	Essence of Mentha piperita (mint) pepper)	61
	Colgate	Total Advanced Healthy Enamel	Essence of Mentha piperita (Peppermint)	62
	Colgate	Total Advanced Healthy Gums	Essence of Mentha piperita (Peppermint)	63
	Colgate	Total Advanced Pure Breath	Essence of Mentha piperita (Peppermint)	64
	Colgate	Total Advanced Deep Cleansing		65
	Colgate	Total Whitener	Essence of Mentha piperita (Peppermint)	66
	Colgate	Total Plaque Protection	Essence of Mentha piperita (Peppermint)	67
	Colgate	Triple Action		68
	Continente	Bi-fluoride		69
	Continente	Whitener		70
	Continente	With coal		71
	Continente	Fresh Gel		72
	Continente	Strawberry flavored children	Strawberry flavor	73
	Continente	Children's bubblegum flavor		74
	Continente	Sensitive		75
	Continente	Triple Action		76
	Corpore Sano	Toothpaste myrrh, propolis and fennel	Foeniculum vulgare (fennel) extract	77
	Couto	couto toothpaste	Essence of Mentha piperita (Peppermint)	78
	Denforsan	Gums Special	Essence of Mentha piperita (Peppermint)	79
	Denforsan	Mint	Fennel	80
	Dentaid	Xeros		81
	Dr. Bronners	Peppermint All-in-one toothpaste	Coconut oil<br>Cocos nucifera (coconut) fruit extract<br>Essence of Mentha piperita (Peppermint)	82
	Dr. Hauschka	Saline toothpaste sensitivity	Essence of Citrus paradisi (grapefruit)<br>Essence of Citrus medica limonum (lemon)	83
	Dr.organic	Extra Whitening Toothpaste Activated Charcoal	Essence of Mentha piperita (Peppermint)	84
	Dr.organic	Purifying Toothpaste Organic Tea Tree	Essence of Mentha piperita (Peppermint)	85
	Dr.organic	Organic Aloe Vera Whitening Toothpaste	Essence of Mentha piperita (Peppermint)	86

	Dr.organic	Whitening Toothpaste Organic Pomegranate	Punica granatum (pomegranate) extract	87
	Fluocaril	Menthol toothpaste		88
	Georganics	Natural Toothpowder Spearmint		89
	Georganics	Natural Toothpowder Tea Tree	Essence of Cinnamomum Zeylanicum Bark (cinnamon)	90
	Georganics	Toothpaste Tablets English Peppermint	Essence of Mentha piperita (Peppermint)	91
	Golden Silk	Natural clay and fennel toothpaste	Essence of Foeniculum vulgare (fennel)	92
	Green Tailors	Toothpaste in tablet form		93
	GUM	ActiVital	Pomegranate<br>Ginger	94
	GUM	Hydral		95
	GUM	Ortho	Ginger	96
	GUM	OriginalWhite		97
	GUM	Paroex		98
	GUM	SensiVital+		99
	Himalaya Herbals	Toothpaste neem and pomegranate	Punica granatum (pomegranate) extract	100
	Homéocaryl	Lemon-scented toothpaste		101
	Homéocaryl	Chlorophyll-scented toothpaste		102
	Hubner	Silicea Toothpaste with Peppermint oil / menthol	Essence of Mentha piperita (Peppermint)<br>Essence of Foeniculum vulgare (fennel)<br>Essence of Eugenia caryophyllus (clove)	103
	Intersa	Aprolis mouthwash	Essence of Mentha piperita (Peppermint)<br>Thymus vulgaris (thyme) extract	104
	ISDIN	Bexident Anti-cáries	Cranberry extract	105
	ISDIN	Bexident Whitener		106
	ISDIN	Bexident Sensitive Teeth		107
	ISDIN	Bexident Gums		108
	ISDIN	Bexident Post		109
	Jasön	Healthy Mouth Anti-cavity & Tartar Control Gel	Carum Petroselinum Extract (parsley)<br>Citrus Grandis (grape) extract<br>Essence of Cinnamomum Zeylanicum Bark (cinnamon)<br>Essence of Eugenia caryophyllus (clove)	110

	Jasön	Healthy Mouth Tartar Control Paste	Essence of Mentha piperita (Peppermint)<br>Carum Petroselinum Extract (parsley)<br>Citrus Grandis (grape) extract<br>Essence of Cinnamomum Zeylanicum Bark (cinnamon)<br>Essence of Eugenia caryophyllus (clove)	111
	Jasön	Power Smile Whitening Toothpaste	Essence of Mentha piperita (Peppermint)<br>Carum Petroselinum Extract (parsley)<br>Citrus Grandis (grape) extract	112
	Jasön	Sea Fresh Strenghtning Paste	Carum Petroselinum Extract (parsley)<br>Citrus Grandis (grape) extract	113
	Jordan	Clinic Gentle Repais		114
	Jordan	Green Clean Cavity Protection	Maltodextrin	115
	Jordan	Junior 6 - 12 years old		116
	Kemphor	Bleaching machine		117
	Kemphor	Charcoal		118
	Kemphor	Fresh		119
	Kemphor	Junior	Strawberry flavor	120
	Kemphor	Natural care		121
	Kemphor	Natural protect	Ginger flavor	122
	Kemphor	Natural white - tea tree and mint		123
	Kemphor	Original		124
	Kemphor	Ortho		125
	Kemphor	Sensitive		126
	Kemphor	White		127
	KIN	SensiKIN Dental Sensitivity		128
	L'Angelica	Total protection toothpaste	Basil	129
	Logodent	Extra fresh Organic Mint	Essence of Mentha piperita (Peppermint)	130
	Logodent	Calcium minerals without fluoride	Essence of Mentha piperita (Peppermint)<br>Essence of Eugenia caryophyllus (clove)	131
	Logodent	Sensitive Chamomile		132
	Logodent	Kids	Maltodextrin<br>Strawberry flavor	133
	Merci Handy	Smile detox	Coconut oil	134
	NaturaBIO cosmetics	Extra fresh echinacea & mint	Essence of Mentha piperita (Peppermint)	135
	Neobio	Children's toothpaste	Carica papaya extract<br>Malus domestica (apple) extract	136
	Neobio	Toothpaste with organic witch hazel and organic rosemary	Rosmarinus Officinalis Essence (rosemary)	137
	Oral-B	1-2-3		138

	Oral-B	3D White Arctic Whiteness		139
	Oral-B	3D White Whitening sensitive teeth		140
	Oral-B	3D White Pearl Effect		141
	Oral-B	3D White Luxe Perfection		142
	Oral-B	Complete + Oral Elixir		143
	Oral-B	Pro-Expert Healthy White		144
	Parodontax	Without whitener		145
	Pierre Fabre - Oral Care	Arthrodont CLASSIC	Essence of Mentha piperita (mint) pepper)	146
	Pierre Fabre - Oral Care	Arthrodont EXPERT		147
	Pierre Fabre - Oral Care	Arthrodont PROTECT		148
	Pierre Fabre - Oral Care	Elgydium Anti-platelet		149
	Pierre Fabre - Oral Care	Elgydium Brilliance & Care		150
	Pierre Fabre - Oral Care	Elgydium Clinic Protection Erosion		151
	Pierre Fabre - Oral Care	Elgydium Clinic Sensileave		152
	Pierre Fabre - Oral Care	Elgydium Fluorinol Sensitive Teeth		153
	Pierre Fabre - Oral Care	Elgydium Junior		154
	Pierre Fabre - Oral Care	Elgydium Kids 500 ppm fluoride	Banana flavor	155
	Pierre Fabre - Oral Care	Elgydium Kids 1000 ppm fluoride	Strawberry flavor	156
	Pierre Fabre - Oral Care	Elgydium Multi-action	Maltodextrin	157
	Pierre Fabre - Oral Care	Elgydium Phyto Fluorinol		158
	Pierre Fabre - Oral Care	Elgydium Plaque & Gums		159
	Pierre Fabre - Oral Care	Elgydium Caries Prevention		160
	Pierre Fabre - Oral Care	Elgydium Gum Protection		161

	Pierre Fabre - Oral Care	Elgydium Whitening		162
	Pierre Fabre - Oral Care	Elgydium Whitening Cool Lemon		163
	Sante	Dental Med Toothpaste Vitamin B12		164
	Sensodyne	Full Action		165
	Sensodyne	Extra Fresh		166
	Sensodyne	Herbal	Essence of Foeniculum vulgare (fennel)	167
	Sensodyne	Multicare		168
	Sensodyne	Pro-Esmalte		169
	Sensodyne	Pro-Esmalte 6 - 12 years		170
	Sensodyne	Daily Protection		171
	Sensodyne	Rapid Action		172
	Sensodyne	Repair & Protect Whitening		173
	Sensodyne	Sensitivity and Gums		174
	Sensodyne	Sensitivity and Gums + Whitening		175
	SriSri	Fluoride-free Ayurvedic toothpaste	Coconut Glucoside<br>Piper nigrum (black pepper) extract<br>Essence of Eugenia caryophyllus (clove)<br>Cinnamomum Zeylanicum Essence Bark (cinnamon)	176
	The Humble Co.	Kids with fluoride		177
	The Humble Co.	Charcoal Toothpaste	Essence of Mentha piperita (Peppermint)	178
	The Humble Co.	Toothpaste Fresh Mint	Essence of Mentha piperita (Peppermint)	179
	Theramed	Junior 6+ years	Pyrus Malus (apple) extract	180
	VITIS	Junior	Watermelon Flavor	181
	VITIS	Junior	Orange Flavor	182
	VITIS	Junior	Kiwi flavor	183
	VITIS	Kids	Cherry aroma	184
	Weleda	Marigold toothpaste		185
	Weleda	Children's toothpaste	Essence of Prunus amygdalus dulcis (sweet almond)	186
	Weleda	Saline toothpaste	Lactose<br>Essence of Mentha piperita (Peppermint)<br>Prunus spinosa (sloe) juice<br>Guar gum (E412) - *Cyamopsis tetragonoloba*	187

**Mouthwashes**	Aquafresh	Junior 6 - 12 years old fruit flavor		188
	Aquafresh	Multi Action Fresh Mint		189
	Ben&Anna	Natural mouthwash		190
	Buccotherm	Alcohol-free	Vaccinium macrocarpon extract (cranberry)	191
	Colgate	Max White		192
	Colgate	Plax Soft Mint		193
	Colgate	Plax Tea & Lemon		194
	Continente	Anti-carious Mouthwash		195
	Continente	Whitening Mouthwash		196
	Continente	Total Care Mouthwash		197
	Continente	Children's Elixir Strawberry flavor	Strawberry flavor	198
	Corpore Sano	Elixir mouthwash myrrh, propolis and fennel	Essence of Foeniculum vulgare (fennel)	199
	Denforsan	Elixir oral hygiene	Essence of Foeniculum vulgare (fennel)	200
	Dentaid	Perio-aid		201
	Dr.organic	Oral Elixir malaleuca bio	Citrus grandis (grape) extract	202
	Eliah Sahil	Tooth oil healthy gums and oral flora	Curcuma xanthorrhiza (Turmeric) extract<br>Essence of Mentha piperita (Peppermint)	203
	Elmex	Caries protection		204
	Elmex	Sensitive professional		205
	Gengigel	Gengigel Mouthwash		206
	Georganics	Oil Pulling Mouthwash Activated Charcoal	Essence of Mentha piperita (Peppermint)<br>Extra Virgin Coconut Essence	207
	Georganics	Oil Pulling Mouthwash English Peppermint	Essence of Mentha piperita (Peppermint)<br>Extra Virgin Coconut Essence	208
	GUM	ActiVital	Punica granatum (pomegranate) extract<br>Zinziber officinale (ginger) extract	209
	GUM	Aphtha Clear	Apple aroma	210
	GUM	Aphtha Clear	Pear flavor	211
	GUM	HaliControl		212
	GUM	Hydral		213
	GUM	Ortho	Zinziber officinale (ginger) extract	214
	GUM	Paroex		215
	Halita	Halita		216
	Ifigen	Rye Mouthwash	Rosmarinus officinalis (rosemary) extract<br>Thymus vulgaris (thyme) extract	217
	ISDIN	Bexident anti-caries	Vaccinium macrocarpon (cranberry) extract	218

	ISDIN	Bexident whitening		219
	ISDIN	Bexident sensitive teeth daily care		220
	ISDIN	Bexident sensitive teeth maintenance		221
	ISDIN	Bexident gums daily care		222
	ISDIN	Bexident intensive care gums		223
	ISDIN	Bexident fresh breath		224
	ISDIN	Bexident post	Chitosan (crustacean cytoskeleton)	225
	Jasön	Elixir Power Smile	Grapefruit extract<br>Papaya extract<br>Essence of Mentha piperita (Peppermint)	226
	Kemphor	Oral Antiseptic		227
	L'Angelica	Anti-plaque mouthwash		228
	Listerine	Advanced White - mild flavor		229
	Listerine	Alcohol-free menthol - mild flavor		230
	Listerine	Tooth & Gum Protection		231
	Listerine	Tooth & Gum Protection - mild flavor		232
	Listerine	Total Care		233
	Oral-B	3D White		234
	Oral-B	Pro-Expert Deep Cleansing		235
	Oral-B	Pro-Expert Professional Protection		236
	Parodontax	Daily gum care		237
	Pierre Fabre - Oral Care	Arthrodont	Essence of Mentha piperita (Peppermint)	238
	Pierre Fabre - Oral Care	Elgydium Junior	Red fruit aroma	239
	Pierre Fabre - Oral Care	Eludril Care		240
	Pierre Fabre - Oral Care	Eludril Classic		241
	Pierre Fabre - Oral Care	Eludril Extra		242
	Pierre Fabre - Oral Care	Eludril Gums		243
	Pierre Fabre - Oral Care	Eludril Intense		244
	Pierre Fabre - Oral Care	Eludril Sensitive		245
	Pierre Fabre - Oral Care	Eludril White		246
	Pierre Fabre - Oral Care	EluPerio		247
	Sensodyne	Cool Mint		248

	The Humble Co.	Natural Coal Elixir	Essence of Mentha piperita (Peppermint)<br>Lycium Barbarum (goji) extract<br>Panax ginseng extract (ginseng)	249
	VITIS	Anticaries		250
	VITIS	Gingival		251
	VITIS	Orthodontic	Apple aroma	252
	VITIS	Whitening		253
**Oral gels**	Abaca	Oroben	Essence of Mentha piperita (Peppermint)	254
	Buccotherm	First Teeth 0 - 2 years		255
	GUM	Aphtha Clear		256
	GUM	Hydral		257
	ISDIN	Bexident Canker sores		258
	ISDIN	Bexident Sensitive Teeth		259
	ISDIN	Bexident Gums Intense Care		260
	ISDIN	Bexident Post	Chitosan (crustacean cytoskeleton)	261
	KIN	PerioKIN	Rosmarinus Officinalis Essence (rosemary)	262
	Mitosyl	basiclsamo first teeth - gingival gel	Milk Protein	263
	Pierre Fabre - Oral Care	Elgydium Clinic Cicalium Gel		264
	Pierre Fabre - Oral Care	Elgydium Clinic Sensileave Gel		265
	Pierre Fabre - Oral Care	Elgydium Repair		266
	Pierre Fabre - Oral Care	Elugel Purifying		267
	Pierre Fabre - Oral Care	Pansoral first baby teeth		268
	Pierre Fabre - Oral Care	Parodium Sensitive Gums	Maltodextrin	269
	Weleda	Sage gel for gums		270
**Oral sprays**	Buccotherm	100% Natural Dental Spray		271
	Buccotherm	Oral Spray Organic Certified	Essence of Mentha piperita (Peppermint)	272
	Dentaid	Xeros Spray		273
	GUM	Hydral		274
	ISDIN	Bexident gums		275
	ISDIN	Bexident fresh breath		276
	ISDIN	Bexident canker sore treatment		277
	Pierre Fabre - Oral Care	Elgydium Clinic Cicalium Spray		278
	Pierre Fabre - Oral Care	Elgydium Clinic Xeroleave Spray		279

**Denture fixative creams**	Continente	Prosthesis fastener		280
	Kukident PRO	Adhesive cream for prosthetics - classic flavor		281
	Polident	Fixing cream - strong hold		282
	Polident	Fixing cream - gum protection		283
**Denture cleaning tablets**	Continente	Effervescent tablets for prosthetics dental		284
	Corega	Total Action Cleaning Pads		285
	Polident	Cleaning tablets with whitener		286
	Polident	Cleaning tablets with active oxygen		287
**Chewing gum**	Buccotherm	Sugarless patties with sweetener	Soy lecithin<br>Arabic gum (E414)	288
	Listerine	Go! Tabs		289
	Pierre Fabre - Oral Care	Elgydium Breath	Parsley Essential Oil	290
	Pierre Fabre - Oral Care	Elgydium anti-plaque chewing gum bacterial - intense freshness		291
	The Humble Co.	Xylitol chewing gum, sugar-free - lemon	Traces of soy<br>Arabic gum (E414)	292
	The Humble Co.	Xylitol chewing gum, sugar-free - mint	Traces of soy<br>Arabic gum (E414)	293
	The Humble Co.	Xylitol chewing gum, sugar free - tropical	Traces of soy and nuts<br>Arabic gum (E414)	294
	Xyligum	Peppermint	Arabic gum (E414)	295
	GC	Tooth Mousse	Casein<br>Vanilla flavor	296
**Topical creams**	GC	MI Paste Plus	Casein<br>Vanilla flavor	297
**Fluoride varnishes**	3M ESPE	Clinpro White Varnish	Caramel aroma	298
	3M ESPE	Clinpro White Varnish	Melon Aroma	299
	3M ESPE	Clinpro White Varnish	Cherry aroma	300
	3M ESPE	Clinpro White Varnish	Raspberry flavor	301
	Colgate	Duraphat		302
	Dentsply Sirona	Nupro	Grape aroma	303
	Dentsply Sirona	Nupro	Raspberry flavor	304
	DMG	Flairesse	Melon Aroma	305
	GC	MI Varnish	Casein	306
	Henry Schein	Fluoride-containing dental desensitising. varnish	Cherry aroma	307
	Ivoclar Vivadent	Cervitec F		308
	Ultardent	Enamelast	Strawberry flavor<br>Amaretto flavor (liqueur made from infused apricots and peaches)<br>Orange Essence	309
	VOCO	Profluorid Varnish lacquer - single use	Caramel aroma	310

	VOCO	Profluorid Varnish lacquer - single use	Cherry aroma	311
	VOCO	Profluorid Varnish lacquer - single use	Melon Aroma	312
	VOCO	Profluorid Varnish lacquer - single use	Pineapple flavor	313
	VOCO	Profluorid Varnish lacquer - single use	Melon Aroma	314
	VOCO	Profluorid Varnish - ampoules	Melon Aroma	315
	VOCO	Profluorid Varnish - paste	Caramel aroma	316
	VOCO	Profluorid Varnish - paste	Melon Aroma	317
	VOCO	Profluorid Varnish - paste	Mint flavor	318
	VOCO	Profluorid Varnish - paste	Cherry aroma	319
	VOCO	Profluorid Varnish - paste	Pineapple flavor	320
**Alginates**	Bestdent	Alginate fast	Orange Flavor	321
	Cavex	CA37 Normal set		322
	Cavex	ColorChange		323
	Cavex	Cream Alginate	Strawberry flavor	324
	Cavex	Impressive		325
	Chromalg'x	Alginate	Raspberry flavor	326
	Cromatic	Cromatic	Vanilla flavor	327
	Cybertech	Ortho		328
	Dentsply Sirona	Blueprint Xcreme		329
	GC	Aroma Fine Plus	Peppermint flavoring	330
	Henry Schein	Krom		331
	INIBSA	Algisul		332
	Kulzer	Alginoplast		333
	Kulzer	Xantalgin Crono fast set		334
	Kulzer	Xantalgin Select fast set		335
	Lascod	Kromopan		336
	Major	Alginkid	Orange Flavor	337
	Major	Alginmajor	Tutti frutti aroma	338
	Major	Alginmax	Vanilla flavor	339
	Medicaline	Algiline: alginate universal fast set	Fruit aroma	340
	Pierre Roland	Chromalg'x	Raspberry flavor	341
	Pierre Roland	Orthofast	Peach flavor	342
	Proclinic	Chromatic	Vanilla flavor	343
	QMS	Bestprint		344
	Vidu	Chromatic Alginate		345
	R&S	Turboprint Chroma	Tropical fruit aroma	346
	R&S	Turboprint Chroma	Citrus aroma	347

	R&S	Turboprint Class A		348
	R&S	Turboprint Ortho	Strawberry flavor	349
	R&S	Turboprint Ortho speed	Tutti frutti aroma	350
	Septodont	Plastalgin	Apple aroma	351
	Septodont	Plastalgin ortho	Apricot flavor	352
	Vannini	Clipalgin		353
	Vannini	Kromalgin	Peppermint flavoring	354
	Zhermack	Hydrogum 5	Tropical fruit aroma	355
	Zhermack	Orthoprint	Vanilla flavor	356
	Zhermack	Phase plus		357
	Zhermack	Neocolloid		358
	Zhermack	Tropicalgin	Mango Aroma	359
**Orthodontic waxes**	Carmel	Protective wax	Pineapple flavor	360
	Ortho Technology	Taste Protection Wax for Orthodontic Patients	Chocolate flavor	361
	Ortho Technology	Taste Protection Wax for Orthodontic Patients	Pineapple flavor	362
	Reus	Mentholated Orthodontic Wax		363
**Plaque revealing cream**	Angelus	Angie Plate Evidence		364
	EMS	Biofilm developer		365
	GC	Tri Plaque ID Gel		366
	Hager Werken	Mira-2-Ton	Lactose	367
	Pierre Fabre - Oral Care	Elgydium plaque developer		368
	TEPE	Plaq-search (tablets)		369
**Prophylactic toothpastes**	Bestdent	Prophylaxis paste	Strawberry flavor	370
	Dentsply	Nupro Prophylaxis	Orange Flavor	371
	Direct	Prophy Paste CSS		372
	Direct	Prophy Paste Pro		373
	Kerr Endo	Cleanic	Apple aroma	374
	Kerr Endo	Cleanic	Red fruit aroma	375
	Clarben Laboratories	Profex	Cherry aroma	376
	R&S	Polishing Paste		377
	Septodont	Détartrine 100ZF		378
	Technew	New prophy paste	Orange Flavor	379
	VOCO	Clean Joy	Caramel aroma	380
	VOCO	Clean Joy	Cherry aroma	381
**Gloves**	Comfort	Latex gloves with powder		382
	Cybertech	Powderless Latex Gloves	Oats (coating)	383

	Dermatex	Powderless Latex Gloves	Oats (coating)	384
	Medistock	Vinyl gloves with powder		385
	Medline	Powderless nitrile gloves	Oats (coating)	386
	Perfectgloves	Powderless nitrile gloves	Oats (coating)	387
